# Efficacy of Intralesional Platelet-Rich Plasma in Patients With de Quervain’s Tenosynovitis

**DOI:** 10.7759/cureus.79617

**Published:** 2025-02-25

**Authors:** Muhammad Tahir Iqbal, Muhammad Zarak Awais, Raja Muhammad Mussab

**Affiliations:** 1 Orthopedic Surgery, King Edward Medical University, Lahore, PAK; 2 Orthopedics and Trauma, Jinnah Postgraduate Medical Centre, Karachi, PAK

**Keywords:** de quervain’s tenosynovitis, diabetes mellitus, efficacy, pain, platelet rich plasma, visual analog scale

## Abstract

Background

De Quervain’s tenosynovitis is a disorder of the tendons of the first dorsal compartment of the wrist that causes pain and functional disability and may be refractory to conservative treatments. This research was done to determine the efficacy of intralesional platelet-rich plasma (PRP) in patients with de Quervain’s tenosynovitis.

Methodology

This quasi-experimental study was performed at the Department of Orthopedic Surgery, King Edward Medical University/Mayo Hospital, Lahore, Pakistan, from September 2021 to October 2022. Patients of either gender, aged between 18 and 60 years, and presenting with de Quervain’s tenosynovitis were analyzed. The patient received a PRP injection plus an oral nonsteroid anti-inflammatory drug (NSAID; naproxen 500 mg twice daily). The patient was evaluated three weeks after treatment for efficacy. Efficacy was labeled as a visual analog scale (VAS) pain score of < 3 after three weeks of intralesional PRP injection.

Results

In a total of 67 patients, 42 (62.7%) were male. The mean age was 44.26±6.48 years. The mean pre-treatment VAS score was 7.24±1.36. The mean post-treatment VAS score after 3 weeks of intralesional PRP injection was 2.14±0.63 (p<0.001). Patients with diabetes had higher post-treatment VAS scores (2.33±0.75) compared to those without (1.99±0.48), indicating a significant difference (p=0.025). Other characteristics, such as hypertension (p=0.334), family history of musculoskeletal disorders (p=0.704), duration of symptoms (0.346), hand dominance (p=0.516), and occupation (p=0.649), did not show significant differences in post-treatment VAS scores. Efficacy was achieved in 85.1% (n=57) of patients. Non-efficacy was significantly associated with diabetes mellitus (80% of non-responders vs. 36.8% of responders, p=0.011). Other factors, such as gender (p=0.289), age (p=0.279), BMI (p=0.258), residence (p=0.933), smoking history (p=0.358), hypertension (p=0.808), family history of musculoskeletal disorders (p=0.553), symptom duration (p=0.983), hand dominance (p=0.096), or occupation (p=0.935), were not significantly associated with treatment efficacy.

Conclusion

Intralesional PRP injections provide effective pain relief in the majority of patients with de Quervain’s tenosynovitis. While the treatment appears effective across most patient subgroups, diabetes mellitus significantly reduces the likelihood of success, indicating the need for personalized treatment approaches in such cases.

## Introduction

De Quervain’s tenosynovitis (DQT) was originally identified by Swiss surgeon Fritz de Quervain in 1895 [1]. It is a condition involving the entrapment of tendons within the first dorsal compartment of the wrist. In DQT, the sheaths surrounding the abductor pollicis longus (APL) and extensor pollicis brevis (EPB) tendons thicken as they pass through a fibro-osseous tunnel near the radial styloid of the distal wrist. This thickening and effusion leads to pain, which worsens with thumb movements and radial or ulnar deviation of the wrist [2]. The underlying cause of DQT is often linked to repetitive stress and overuse of the APL and extensor pollicis brevis (EPB) tendons, leading to inflammation and thickening within the first dorsal compartment. This may result in increased friction and restriction of tendon gliding, contributing to pain and dysfunction.

Patients commonly report pain and swelling near the radial styloid, which becomes more pronounced during activities that involve ulnar deviation with the thumb's metacarpophalangeal (MP) joint flexed such as wringing out a cloth, gripping a golf club, lifting a child, or hammering. Continued engagement in these activities tends to exacerbate the inflammation [3]. Diagnosis is typically clinical, though plain radiographs can help rule out other potential causes of radial wrist pain such as thumb carpometacarpal joint osteoarthritis [4,5]. DQT is sometimes self-limiting, but for patients with persistent symptoms, conservative treatments, such as splinting, anti-inflammatory medications, and corticosteroid injections, are often employed. Using a thumb spica splint may provide short-term relief, though compliance can be low and recurrence rates are often high [6,7].

Platelet-rich plasma (PRP) therapy has emerged as a treatment option, as it involves the use of an autologous concentration of growth factors and inflammatory mediators [8,9]. In a study by Ramesh et al., PRP was found to be 95.5% effective in treating DQT [10]. In another study done by Bender et al., the mean visual analog scale (VAS) pain scores decreased by an average of 87% after PRP therapy in DQT [11].

Traditional treatment methods, such as corticosteroid injections and physical therapy, often provide only temporary relief and may not address the underlying inflammation effectively. Intralesional PRP therapy has emerged as a promising alternative, utilizing the patient's own growth factors and cytokines to promote healing and reduce inflammation. By investigating the efficacy of intralesional PRP in patients with DQT, this research aims to provide evidence for a minimally invasive treatment option that could enhance recovery times, reduce pain, and improve functional outcomes. This study aimed to determine the efficacy of intralesional PRP in patients with DQT.

## Materials and methods

This quasi-experimental study was performed at the Department of Orthopedic Surgery, Kind Edward Medical University/Mayo Hospital, Lahore, Pakistan from September 2021 to October 2022. A sample size of 67 was calculated taking the expected efficacy of PRP in DQT as 95.5%, with a 5% margin of error and a 95% confidence level [10]. The non-probability consecutive sampling technique was adopted. Approval from the Institutional Ethical Committee of Kind Edward Medical University/Mayo Hospital, Lahore, Pakistan was obtained (letter number: KEMU/2021/RC/67). Informed as well as written consent was obtained. This clinical trial was registered with reference number NCT06651034 at clinicaltrials.gov.

The inclusion criteria were patients of either gender, aged between 18 and 60 years, and presenting with DQT. Exclusion criteria were patients having previous surgery or intervention for DQT or those who had a local skin infection or osteomyelitis detected by clinical and radiological examination. Pregnant or lactating mothers were also not included. DQT was diagnosed with a history of pain of more than 3 on VAS in the wrist and thumb for more than one month and having a positive Finkelstein test (aggravation of pain > 2 points on VAS) on flexing the thumb in the palm with simultaneous ulnar deviation of the wrist. Under aseptic conditions, 20 mL of venous blood was collected from each patient using sterile sodium citrate anticoagulant vacutainer tubes. The blood was processed using Eppendorf 5702. A double-spin centrifugation technique was employed. The first spin at 1500 RPM for 10 minutes to separate red blood cells from plasma. The second spin at 3000 RPM for 10 minutes to concentrate the platelets. After centrifugation, the middle buffy coat layer, rich in platelets, was carefully aspirated into a 5 mL sterile syringe. The final PRP volume obtained was approximately 3-5 mL, ensuring a high platelet concentration while minimizing red and white blood cell contamination. The intralesional PRP injection was administered at the site of maximal tenderness under sterile conditions. Oral Naproxen 500 mg twice daily was prescribed for symptomatic relief for a duration of two weeks. Patients were followed up at three weeks post-treatment to evaluate efficacy. Efficacy was labeled as VAS pain score < 3 after three weeks of intralesional PRP injection.

Data were entered and analyzed in SPSS, version 26.0 (IBM Corp., Armonk, NY, US). Quantitative variables like age, BMI, and pre and post-treatment VAS scores were calculated as mean along with standard deviation (SD). Qualitative variables like gender, diabetes mellitus, and efficacy were shown as frequencies and percentages. Data were stratified for age, BMI, gender, and diabetes mellitus, with respect to efficacy, and a post-stratification chi-square test was applied taking p < 0.05 as the level of statistical significance.

## Results

In a total of 67 patients, 42 (62.7%) were male. The mean age was 44.26±6.48 years. The mean BMI was 26.49±2.08 kg/m^2 ^while 43 (64.2%) patients had a BMI >25 kg/m². Residential affiliations of 41 (61.2%) patients were rural. History of smoking was present in 14 (20.9%), diabetes mellitus in 29 (43.3%), and hypertension in 18 (26.9%). The duration of symptoms was ≥6 months in 40 (59.7%) patients. The mean pre-treatment VAS pain scores ≥6 were noted in 82.1% of patients. The mean pre-treatment VAS score was 7.24±1.36. Table 1 shows details about the distribution of baseline demographical and clinical characteristics of the patients.

**Table 1:** Baseline demographical and clinical characteristics (n=67)

Characteristics		Frequency (%)
Gender	Male	42 (62.7%)
	Female	25 (37.3%)
Age (years)	18-35	7 (10.4%)
	36-60	60 (89.6%)
Body mass index (kg/m^2^)	17-25	24 (35.8%)
	>25	43 (64.2)
Residence	Urban	26 (38.8%)
	Rural	41 (61.2%)
History of smoking		14 (20.9%)
Diabetes mellitus		29 (43.3%)
Hypertension		18 (26.9%)
Family history of musculoskeletal disorders		11 (16.4%)
Duration of symptoms (months)	<6	27 (40.3%)
	≥6	40 (59.7%)
Hand dominance	Right	58(86.9%)
	Left	9 (13.4%)
Occupation	Manual labor	17 (25.4%)
	Office worker	29 (43.3%)
	Others	21 (31.3%)
VAS pain score	<6	12 (17.9%)
	≥6	55 (82.1%)

The mean post-treatment VAS score after three weeks of intralesional PRP injection was 2.14±0.63. There were no significant differences in post-treatment pain scores between gender (p=0.382), age groups (p=0.787), BMI categories (p=0.632), or smoking history (p=0.918). Patients with diabetes had higher post-treatment VAS scores (2.33±0.75) compared to those without (1.99±0.48), indicating a significant difference (p=0.025). Other characteristics, such as hypertension (p=0.334), family history of musculoskeletal disorders (p=0.704), duration of symptoms (0.346), hand dominance (p=0.516), and occupation (p=0.649), did not show significant differences in post-treatment VAS scores. Table 2 shows a comparison of post-treatment VAS scores with respect to the baseline characteristics of the patients.

**Table 2:** Comparison of post-treatment VAS score with respect to baseline demographical and clinical characteristics (n=67) VAS: visual analog scale

Characteristics		Post-treatment VAS pain scores	P-value
Gender	Male	2.09±0.57	0.382
	Female	2.23±0.72	
Age (years)	18-35	2.20±0.74	0.787
	36-60	2.13±0.62	
Body mass index (kg/m^2^)	17-25	2.09±0.38	0.632
	>25	2.17±0.74	
Residence	Urban	2.03±0.73	0.285
	Rural	2.20±0.56	
History of smoking	Yes	2.12±0.54	0.918
	No	2.14±0.66	
Diabetes mellitus	Yes	2.33±0.75	0.025
	No	1.99±0.48	
Hypertension	Yes	2.02±0.73	0.334
	No	2.18±0.59	
Family history of musculoskeletal disorders	Yes	2.12±0.31	0.704
	No	2.15±0.68	
Duration of symptoms (months)	<6	2.05±0.57	0.346
	≥6	2.20±0.67	
Hand dominance	Right	2.12±0.61	0.516
	Left	2.27±0.75	
Occupation	Manual labor	2.11±0.63	0.649
	Office worker	2.22±0.60	
	Others	2.05±0.68	
Baseline VAS pain score	<6	2.25±0.73	0.506
	≥6	2.11±0.61	

Efficacy was achieved in 85.1% (n=57) of patients. Non-efficacy was significantly associated with diabetes mellitus (80% of non-responders vs. 36.8% of responders, p=0.011). Other factors, such as gender (p=0.289), age (p=0.279), BMI (p=0.258), residence (p=0.933), smoking history (p=0.358), hypertension (p=0.808), family history of musculoskeletal disorders (p=0.553), symptom duration (p=0.983), hand dominance (p=0.096), or occupation (p=0.935), were not significantly associated with treatment efficacy, and the details are shown in Table 3.

**Table 3:** Comparison of baseline demographical and clinical characteristics of patients with respect to efficacy (N=67)

Characteristics		Efficacy		P-value
		Yes (n=57)	No (n=10)	
Gender	Male	37 (64.9%)	5 (50.0%)	0.289
	Female	20 (35.1%)	5 (50.0%)	
Age (years)	18-35	5 (8.8%)	2 (20.0%)	0.279
	36-60	52 (91.2%)	8 (80.0%)	
Body mass index (kg/m^2^)	17-25	22 (38.6%)	2 (20.0%)	0.258
	>25	35 (61.4%)	8 (80.0%)	
Residence	Urban	22 (38.6%)	4 (40.0%)	0.933
	Rural	35 (61.4%)	6 (60.0%)	
History of smoking		13 (22.8%)	1 (10.0%)	0.358
Diabetes mellitus		21 (36.8%)	8 (80.0%)	0.011
Hypertension		15 (26.3%)	3 (30.0%)	0.808
Family history of musculoskeletal disorders		10 (17.5%)	1 (10.0%)	0.553
Duration of symptoms (months)	<6	23 (40.4%)	4 (40.0%)	0.983
	≥6	34 (59.6%)	6 (60.0%)	
Hand dominance	Right	51 (89.5%)	7 (70.0%)	0.096
	Left	6 (10.5%)	3 (30.0%)	
Occupation	Manual labor	14 (24.6%)	3 (30.0%)	0.935
	Office worker	25 (43.9%)	4 (40.0%)	
	Others	18 (31.6%)	3 (30.0%)	
VAS pain score	<6	11 (19.3%)	1 (10.0%)	0.479
	≥6	46 (80.7%)	9 (0.0%)	

## Discussion

Our findings indicated that PRP injections were highly effective, with a significant reduction in mean VAS pain scores from 7.24±1.36 pre-treatment to 2.14±0.63 post-treatment. Efficacy, defined as a post-treatment VAS score <3, was achieved in 85.1% of patients. Rahman et al. reported an 85% reduction in pain scores following PRP treatment in a similar group of patients [12]. Wani et al. reported an 85% success rate in their cohort, although their study also included a broader range of musculoskeletal conditions [13]. The PRP seems to demonstrate a notable capacity to reduce pain and improve function, underscoring its potential as an alternative to conservative therapies like corticosteroids or nonsteroidal anti-inflammatory drugs (NSAIDs). The lack of significant differences in post-treatment VAS scores across several demographic variables, such as gender (p=0.382), age (p=0.787), and BMI (p=0.632), suggests that PRP may offer consistent results across diverse patient populations. This is an important finding, as it indicates that PRP’s efficacy is not limited by demographic factors, a conclusion shared by Asaad et al., who also found no significant gender differences in their cohort of patients treated with PRP for DQT [14]. In our study, male and female patients had similar post-treatment VAS scores (2.09±0.57 and 2.23±0.72, respectively), reinforcing the broad applicability of PRP injections in clinical practice.

Our study also explored the role of diabetes mellitus in influencing treatment outcomes. Diabetic patients exhibited higher post-treatment VAS scores (2.33±0.75) compared to nondiabetic patients (1.99±0.48, p=0.025). Diabetes was significantly associated with non-efficacy (p=0.011), with 80% of non-responders being diabetic. These findings are consistent with those of Wani et al., who noted that diabetes mellitus could potentially limit the effectiveness of PRP [13]. This reduced efficacy in diabetic patients is likely due to impaired wound healing and tissue regeneration in diabetes, which may affect the biological mechanisms through which PRP exerts its effects such as stimulating collagen production, angiogenesis, and tissue repair [15]. Shoma et al. also identified diabetes as a variable influencing PRR treatment outcomes, reporting lower functional improvements in diabetic patients compared to non-diabetic ones [16].

Our study examined the influence of other clinical factors on PRP efficacy. No significant differences were found in post-treatment VAS scores based on smoking history (p=0.918), hypertension (p=0.334), family history of musculoskeletal disorders (p=0.704), symptom duration (p=0.346), hand dominance (p=0.516), or occupation (p=0.649). These findings suggest that while PRP appears to be effective across various clinical contexts, the presence of diabetes may be the primary factor limiting its efficacy. Rahman et al. also explored similar factors in their study and concluded that PRP treatment was effective regardless of smoking history or occupation, further supporting our conclusions [12].

One of the key strengths of PRP is its ability to provide sustained pain relief over time. Hidajat et al. conducted a systematic review that pooled data from multiple studies and concluded that PRP injections led to significant reductions in VAS pain scores at one and six months post-treatment compared to conservative treatments [17]. In our study, we observed a marked reduction in pain as early as three weeks post-injection. While our follow-up period was shorter, the rapid pain relief we observed is promising, as it suggests that patients may experience early improvements in function and quality of life, with the potential for longer-term benefits. Asaad et al. emphasized the importance of ultrasound (US)-guided PRP injections for enhancing accuracy and efficacy [14]. In their study, PRP injections were administered under US guidance, leading to significant reductions in retinaculum thickness and tendon sheath effusion, as well as substantial pain relief. Although we did not use US guidance in our study, the efficacy rates we achieved are comparable, suggesting that PRP can be effective even without image guidance. However, incorporating US guidance in future studies may further optimize results by ensuring precise placement of the PRP injection within the affected tendon sheath [18].

The comparison of PRP with corticosteroid (CS) injections is an important area of investigation in musculoskeletal medicine. Several studies, including those by El Sheikh et al. and Giroti et al., have demonstrated that while CS injections may provide more immediate pain relief, PRP offers superior long-term outcomes [19,20]. Giroti et al. reported that PRP led to greater reductions in VAS scores and Disabilities of the Arm, Shoulder, and Hand (DASH) scores at six months compared to CS injections [20]. El Sheikh et al. found that although CS initially reduced pain more effectively, PRP had better outcomes in terms of pain relief and hand function at six months [19].

One limitation of our study was the relatively short follow-up period of three weeks. While we observed significant pain reduction within this timeframe, longer follow-up is needed to assess the durability of PRP’s effects. Rahman et al. and Shoma et al. both conducted six-month follow-up studies and found sustained improvements in pain and function with PRP [11,16]. Extending the follow-up period in future studies would provide a clearer understanding of the long-term benefits of PRP and whether additional injections or maintenance therapies are required to sustain efficacy. Another limitation is the sample size of 67 patients, which, while sufficient for demonstrating significant differences in pain scores and efficacy, limits the generalizability of our findings. Larger studies with diverse populations are needed to validate these results and explore the potential for PRP as a first-line treatment for DQT. Our study focused solely on pain reduction, without evaluating functional outcomes or structural changes in the tendons, which could provide further insight into the regenerative effects of PRP. Despite these limitations, our study contributes valuable data to the growing literature on PRP for musculoskeletal conditions. The high efficacy rates, rapid pain relief, and broad applicability of PRP across different demographic and clinical subgroups suggest that it is a promising treatment for DQT. The significant association between diabetes and non-efficacy highlights the need for further investigation into how comorbidities may affect PRP’s therapeutic potential. One of the limitations was the concomitant use of naproxen for symptomatic relief, which may have influenced pain outcomes. Although naproxen was prescribed on an as-needed basis for two weeks, and the final evaluation of PRP efficacy was conducted at three weeks, the potential residual effects of NSAIDs on inflammation and pain perception cannot be entirely ruled out. NSAIDs have potential antiplatelet effects, which might theoretically interfere with PRP-mediated healing. Future studies with a control group receiving only NSAIDs or a PRP-only treatment arm would help better delineate the independent effects of PRP.

## Conclusions

Intralesional PRP injections provide effective pain relief in the majority of patients with de Quervain’s tenosynovitis. While the treatment appears effective across most patient subgroups, diabetes mellitus significantly reduces the likelihood of success, indicating the need for personalized treatment approaches in such cases. Further research with larger sample sizes, longer follow-up, and functional outcome measures is necessary to confirm PRP’s role as a primary treatment option for DQT and to optimize its use in clinical practice.
